# The New Impact Factor of the Arquivos Brasileiros de Cardiologia (ABC Cardiol), 1.318: An Achievement of the SBC for Our Scientific Community

**DOI:** 10.5935/abc.20180129

**Published:** 2018-07

**Authors:** Carlos Eduardo Rochitte

**Affiliations:** Instituto do Coração (InCor), Hospital das Clínicas da Faculdade de Medicina da Universidade de São Paulo (HCFMUSP), São Paulo, SP – Brazil. Hospital do Coração (HCOR), São Paulo, SP – Brazil

**Keywords:** Periodicals, Journal Impact Factor, Databases as Topic, Editorial Policies

The *ABC Cardiol* is indexed in the main databases, such as ISI Web of Science, Cumulated Index Medicus - MEDLINE, Pubmed Central, EMBASE, SCOPUS, SCIELO and LILACS, and it has obtained an Impact Factor (IF) of 1.318 from JCR, as well as a B2 rating by the CAPES Qualis System. According to the list recently released by the Journal Citation Reports 2018,^1^ 12,271 journals were ranked with a wide IF variability. Of these, 130 journals were in the field of Cardiology and Cardiovascular Sciences, and the *European Heart Journal* leads with 23,425. Around 58% of these publications have IFs below 2.0, among which the *Arquivos Brasileiros de Cardiologia* (*ABC Cardiol*), which has the best IF for journals in the area of Cardiology and Cardiovascular Sciences in Brazil, i.e., 1.318, with a total of 2,541 citations in 2017 (Figure 1).^1^

**Figure 1:**
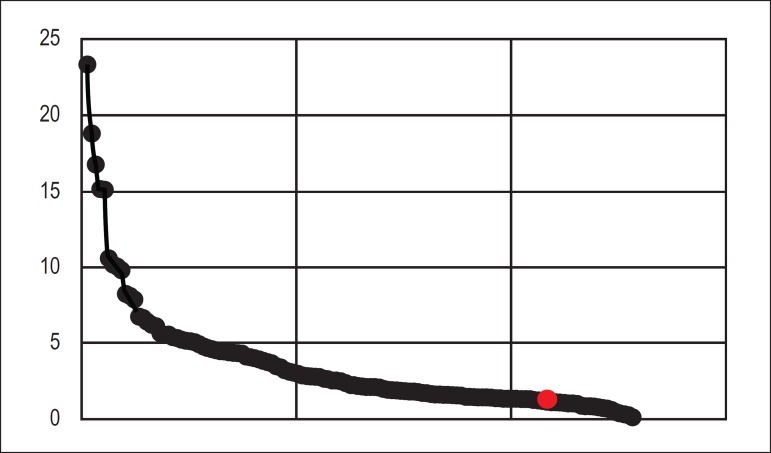
2017 Impact Factor for journals in the area of cardiology and cardiovascular sciences (from JCR). In red, the position of ABC Cardiol. Source: Journal Citation Reports 2018.^1^

There has been a steady increase in *ABC Cardiol*’s IF in the last 5 years (Figure 2A),^1^ resulting from the editorial policies adopted, among which the following stand out: peer-reviewed scientific contributions; members of the Editorial Board and reviewers selected among the most important researchers in Brazil and abroad; the rapid assessment of works which are accepted according to relevance and originality, scientific accuracy and level of importance for the advancement of science; indexing in the main databases; and bilingual open-access publication at no cost for authors. It is worth noting that self-citation was not focused on, as shown by Figure 2B,^2^ which reinforces that the new impact factor is a solid achievement of our scientific community.

**Figure 2:**
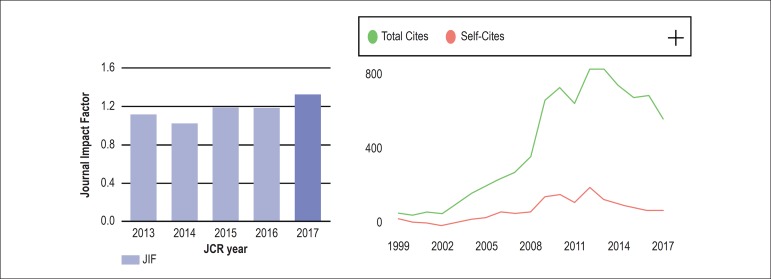
Chart A – Evolution of the ABC Cardiol’s impact factor in the last 5 years. Source Journal Citation Reports 2018.^1^ Chart B – Evolution of total and self-citations. Source: Scimago.^2^

It should be noted that, for the period from 2010 to 2017, original articles were the ones that stood out most in the journal, followed by review articles, both accounting for most of citations (Figure 3). The articles published are divided in 10 areas of knowledge, with 64% of all articles being published in the areas of clinical cardiology, diagnostic methods, basic research and cardiovascular epidemiology (Figure 4), mostly from graduate programs in cardiology, medicine and related areas, which account for almost 60% of the original articles published (Figure 5). Since 2015, the Brazilian Society of Cardiology has annually held a meeting of graduate cardiovascular science program coordinators to discuss the evaluation conducted by CAPES, prospects for journals in the field and internationalization in order to congregate Brazilian researchers with their main national journal.

**Figure 3:**
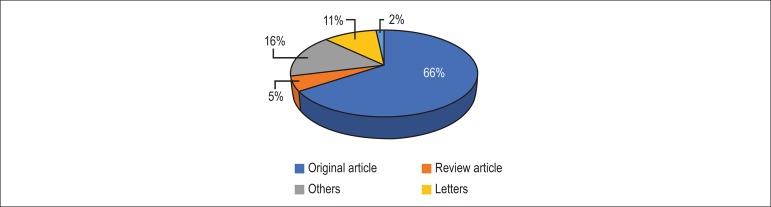
Articles Published (2010-2017).

**Figure 4:**
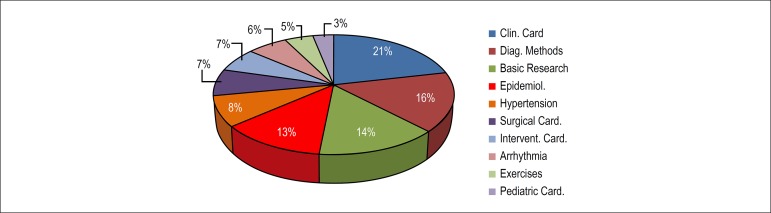
Areas of Knowledge (2010-2017).

**Figure 5:**
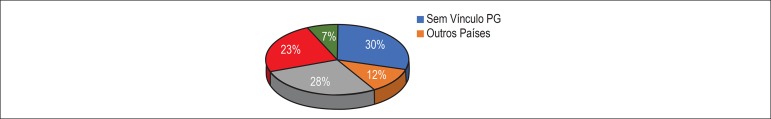
Origin of Publications (2010-2017)

To promote the internationalization of the *ABC Cardiol*, international partnerships were fostered, accounting for 21% of the articles published in 2017, with the U.S., Portugal and Turkey standing out (Figure 6). It is also worth highlighting that 20% of the editorial staff is formed by members linked to foreign institutions. In 2017, the journal received 650 articles for evaluation, 171 of which were approved and 472 rejected, i.e., an approval rate of 26%.

**Figure 6:**
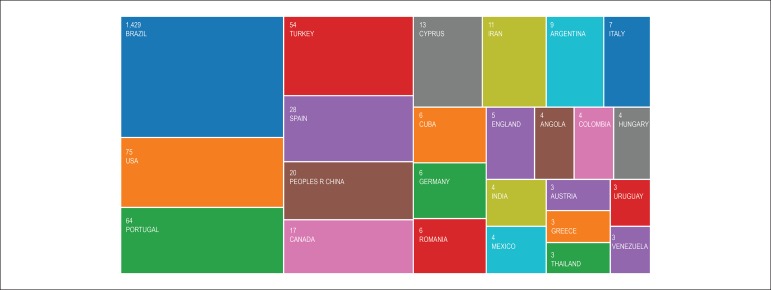
Articles by country (2010-2017). Source: Web of Science.

According to the Web of Science Platform,^3^ the average citation per article was 3.47, with the number of citations increasing each year. Such visibility growth can be attributed in part to the journal’s website and to dissemination in social media. Our site was modernized in 2018 to integrate the SBC’s scientific publications portal,^4^ which had 45,000 visits in 2017. The *ABC Cardiol* keeps a page on Facebook and Twitter,^5,6^ and in April 2018, it created its Instagram page,^7^ where 4 highlight articles from each month’s issue are monthly released, as well as videos of authors and editors, in addition to journal news and campaigns.

However, much remains to be done in order to increase both citations and the journal’s IF. As of June 2018, *ABC Cardiol* began to use the ScholarOne manuscript management system,^8^ thus accepting preprint articles and collecting the authors' ORCid. We also plan to implement Altimetric statistics in the second half of 2018.

This result, which we proudly celebrate, is largely due to the joint efforts of the editors-in-chief, area editors, editorial staff, reviewers and collaborators, who have worked so hard in recent years so that this new IF could be disseminated. Special reference and acknowledgement is made to Prof. Luiz Felipe Moreira, who has led the *ABC Cardiol* on this successful editorial line over the last 8 years. Our thanks to the entire “*ABC Cardiol* family”, as well as to the SBC board members, who have remained faithful to the mission of the Society, which aims to broaden and disseminate knowledge in cardiovascular science, as well as represent and promote the development of Brazilian cardiologists.

## References

[1] InCites: calibrate your strategic research vision.

[2] Scimago Journal & Country Rank (SJR).

[3] (2018). Web of Sciene.

[4] Sociedade Brasileira de Cardiologia (SBC) Publicações Científicas.

[5] Sociedade Brasileira de Cardiologia. (SBC) ABC Cardiol/Journal of Brazilian Society of Cardiology/Facebook.

[6] Sociedade Brasileira de Cardiologia (SBC) ABC Cardiol/Journal of Brazilian Society of Cardiology/Twitter.

[7] Sociedade Brasileira de Cardiologia (SBC) ABC Cardiol/Journal of Brazilian Society of
Cardiology/Instagram.

[8] Clarivate Analytics ScholarOne Manuscripts.

